# *BhSUN* coordinates calcium signaling, microtubule organization, and hormone metabolism to drive directional fruit growth in wax gourd

**DOI:** 10.1093/hr/uhag190

**Published:** 2026-05-12

**Authors:** Zhikui Cheng, Liwen Su, Xialei Huang, Xianglei Chen, Wenjin Yu, Peng Wang, Zhengguo Liu

**Affiliations:** College of Agriculture, Guangxi University, Nanning, Guangxi 530004, China; College of Agriculture, Guangxi University, Nanning, Guangxi 530004, China; College of Agriculture, Guangxi University, Nanning, Guangxi 530004, China; College of Agriculture, Guangxi University, Nanning, Guangxi 530004, China; College of Agriculture, Guangxi University, Nanning, Guangxi 530004, China; College of Agriculture, Guangxi University, Nanning, Guangxi 530004, China; College of Agriculture, Guangxi University, Nanning, Guangxi 530004, China

## Abstract

Fruit shape is a critical determinant of market value in wax gourd (*Benincasa hispida*), yet its genetic and mechanistic basis remains largely unknown. Here, we identify *BhSUN*, which encodes an IQ67-domain (IQD) family protein, as a major regulator of fruit shape through quantitative trait locus mapping in a recombinant inbred line population. *BhSUN* harbors two nonsynonymous single-nucleotide polymorphisms that define haplotypes strongly associated with long cylindrical (*BhSUN*^CC–TT^) vs. spherical (*BhSUN*^GG–GG^) fruits. Near-isogenic line analysis, F₂ segregation, marker-assisted selection, and clustered regularly interspaced short palindromic repeats/CRISPR-associated protein 9 (CRISPR/Cas9)-mediated knockout consistently demonstrate that loss of *BhSUN* function causes a transition from long cylindrical to spherical fruits. Histological analyses reveal that *BhSUN* controls fruit morphology by coordinating oriented cell division and cell expansion. Protein interaction assays show that BhSUN protein interacts with calcium-bound calmodulin (CaM), CaM-like proteins, and the microtubule (MT)-associated protein MAP65-1, thereby linking calcium signaling to MT organization. Concurrently, phytohormone analysis revealed changes in auxin, cytokinin, and gibberellin pathways during *BhSUN*-mediated fruit shape regulation. Our findings identify *BhSUN* as a key regulator that integrates calcium signaling, MT dynamics, and hormone metabolism to direct directional fruit morphogenesis.

## Introduction

Wax gourd (*Benincasa hispida*) is a widely cultivated cucurbit crop of high economic importance, valued for its culinary and medicinal applications [1]. Fruit shape is a key determinant of commercial quality, consumer preference, and end-use diversification, and is therefore considered a major breeding target [2]. Long-term domestication and region-specific market selection have resulted in extensive fruit-shape diversity in wax gourd germplasm, ranging from elongated to nearly spherical forms. In this context, dissecting the genetic basis and regulatory networks underlying fruit-shape formation is essential for precision breeding and genetic improvement in wax gourd.

Fruit shape is commonly quantified using fruit length (FL), fruit diameter (FD), and the fruit shape index (FSI = FL/FD) [3]. Among cucurbit crops, cucumber has emerged as a primary model for elucidating the genetic and molecular mechanisms governing fruit-shape regulation, and its developmental network is relatively well characterized. The major-effect quantitative trait locus (QTL) FS5.2 harbors *CRABS CLAW* (*CsCRC*), a YABBY family gene that activates auxin-responsive protein 1 (*CsARP1*), thereby promoting cell expansion and fruit elongation. This pathway is transcriptionally regulated by *SEPALLATA2* (*CsSEP2*), forming the positive regulatory module CsSEP2–CsCRC–CsARP1 [4–6]. Gain-of-function alleles of the MADS-box gene *FRUITFULL 1* (*CsFUL1*) reshape local auxin distribution through modulation of *SUPERMAN* (*CsSUP*) and auxin transporters *PIN-FORMED1* and *7* (*CsPIN1/7*) expression, collectively suppressing both cell division and cell expansion [7]. The causal gene underlying *FS2.1*, *TONNEAU1 RECRUITING MOTIF* (*CsTRM5*), contributes to fruit morphogenesis by regulating the orientation of cell division [8, 9]. Notably, epistatic interactions between the ethylene biosynthesis gene *1-aminocyclopropane-1-carboxylate synthase 2* (*ACS2*) and the m^6^A reader protein *YTH1* further indicate that RNA modification may also participate in fruit-shape regulation [10].

In addition to these genes, *CsSUN*, located within the *FS1.2* interval, has been implicated in cucumber fruit-shape formation [9, 11]. The *SUN* gene was initially identified in tomato, where family members *SlSUN10* and *SlSUN18* are closely associated with fruit-shape variation [12–15]. SUN proteins belong to the plant-specific IQD family, which is characterized by a highly conserved IQ67 motif [16]. Accumulating evidence indicates that IQD proteins modulate cell morphology and organ development. For instance, the auxin-inducible rice protein *OsIQD14* controls cell shape and grain size, whereas *CaIQD1* in pepper regulates fruit shape through multiprotein complex formation [17, 18]. The IQD family of calmodulin (CaM)-binding proteins links calcium (Ca^2+^) signaling to microtubules (MTs), membrane subdomains, and the nucleus, thereby regulating cellular function, cell shape, and growth [19].

The contribution of *SUN* genes to fruit-shape regulation has also been validated in other cucurbit species. In watermelon, a 159-bp deletion in *ClFS1* (*ClSUN*) underlies the morphological divergence between elongated and spherical fruits, whereas in melon, overexpression of *CmSUN* markedly increases the FSI [20–22]. Although *SUN* genes are widely regarded as strong candidates underlying fruit-shape variation in cucurbits, their molecular mechanisms remain poorly defined, thereby warranting further functional and mechanistic investigation.

In this study, we developed a recombinant inbred line (RIL) population derived from long-cylindrical and spherical wax gourd accessions to perform QTL mapping and identify *BhSUN* as a candidate gene controlling fruit shape. Clustered regularly interspaced short palindromic repeats/CRISPR-associated protein 9 (CRISPR/Cas9)-mediated knockout analysis subsequently confirmed its essential role in fruit-shape determination in wax gourd. Histological analyses revealed that *BhSUN* modulates fruit morphology by regulating both the orientation of cell division and cell expansion. Protein–protein interaction assays further demonstrated that BhSUN directly interacts with Ca^2+^ sensors, including CaM and CaM-like (CML) proteins, as well as the MT-associated protein MAP65-1. In addition, endogenous phytohormone quantification combined with exogenous hormone treatments suggests that *BhSUN* may be associated with the modulation of phytohormone biosynthesis and signaling pathways. Collectively, these findings elucidate a molecular mechanism underlying fruit-shape formation in wax gourd and provide valuable genetic targets for fruit-shape improvement in cucurbit crops.

## Results

### Identification of a major-effect QTL for fruit shape and characterization of the candidate gene *BhSUN*

**Figure 1:**
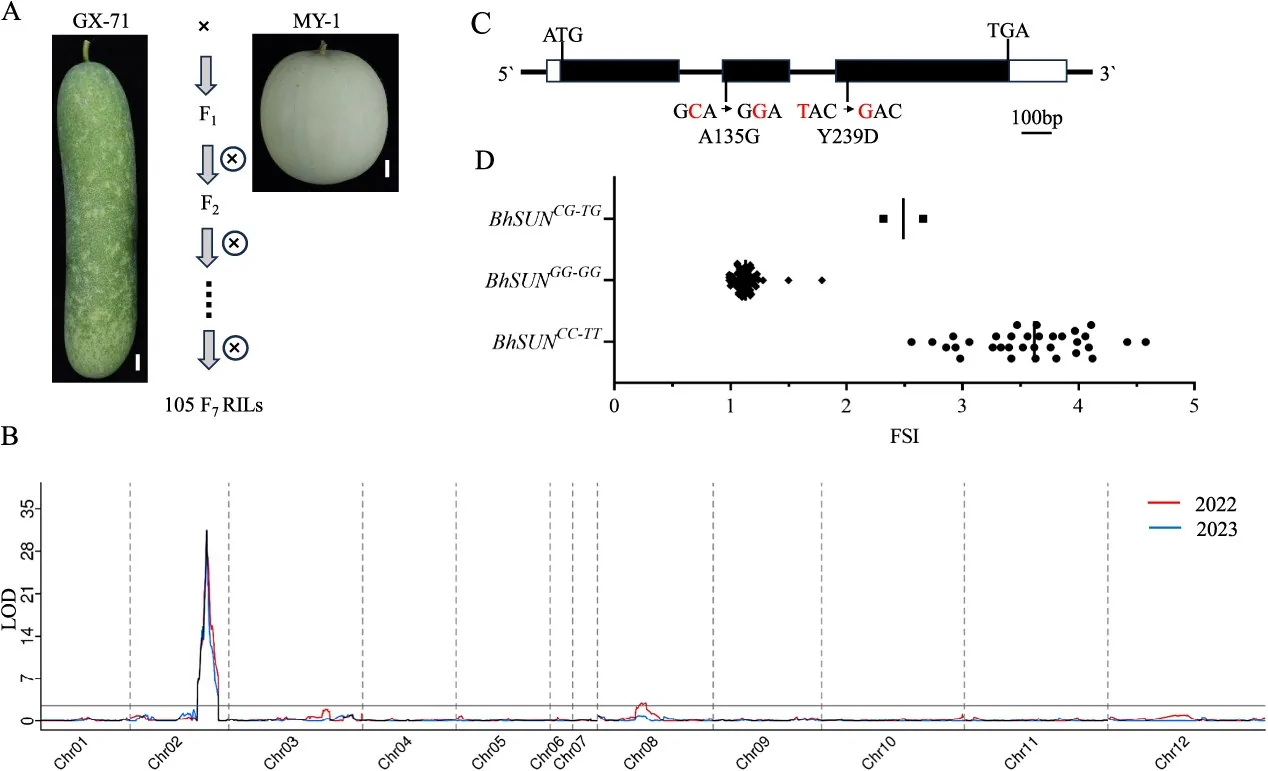
Identification of *BhSUN* as a candidate gene controlling fruit shape in wax gourd. (A) Development of the RIL population derived from a cross between GX-71 (long cylindrical fruit) and MY-1 (spherical fruit). The F₁ plants were self-pollinated for successive generations to generate 105 F_7_ RILs. Representative fruits of the two parental lines are shown. Scale bars = 2 cm. (B) Composite interval mapping of FSI using phenotypic data collected in 2022 and 2023. The logarithm of odds score is plotted against chromosomal position. The horizontal line indicates the significance threshold determined by 1000 permutation tests at *P* = 0.05. (C) Gene structure of *BhSUN* and positions of sequence polymorphisms between the parental lines. Black boxes represent exons, white boxes indicate untranslated regions, and lines denote introns. Two nonsynonymous mutations (A135G and Y239D) are indicated with corresponding nucleotide substitutions. (D) Distribution of FSI among RILs carrying different *BhSUN* haplotypes. Each dot represents an individual RIL, and horizontal lines indicate mean FSI values for each genotype.

To dissect the genetic architecture underlying fruit-shape variation in wax gourd, an RIL population consisting of 105 F_7_ families was developed from a cross between the long-cylindrical-fruit inbred line GX-71 and the spherical-fruit inbred line MY-1. The FSI was evaluated across two consecutive growing seasons (Fig. 1A). Based on the 2-year FSI dataset, composite interval mapping was performed using QTL Cartographer with a high-density genetic linkage map constructed from whole-genome resequencing data.

A stable major-effect QTL was consistently detected on chromosome 2, spanning 57 800 904–58 303 520 bp. This locus exhibited logarithm of odds (LOD) scores of 30.53 and 31.49 in the two respective years and explained 42.80% and 58.40% of the phenotypic variance (Fig. 1B, Table S2). According to the reference genome annotation, the QTL interval contained 14 predicted genes, among which four genes (*Bch02G016720*, *Bch02G016730*, *Bch02G016800*, and *Bch02G016830*) harbored exonic polymorphisms. Notably, *Bch02G016830* carried two nonsynonymous single-nucleotide polymorphisms (SNPs) located in exon 2 (C474G) and exon 3 (T878G) (Fig. 1C). Genotype–phenotype association analysis across the 105 RILs revealed that the CC–TT haplotype of *Bch02G016830* was significantly associated with long cylindrical fruits, whereas the GG–GG haplotype was strongly associated with spherical fruits (Fig. 1D, Table S1). *Bch02G016830* encodes a SUN family protein and shows the highest sequence similarity to the tomato fruit-shape regulator *SlSUN18* (Fig. S1); notably, both polymorphic sites are highly conserved among species (Fig. S2). Collectively, these results identify *Bch02G016830* as the most likely candidate gene underlying fruit-shape variation in wax gourd, and it was therefore designated *BhSUN*.

### Phenotypic effects of natural *BhSUN* variation and fruit-shape divergence in near-isogenic lines

**Figure 2:**
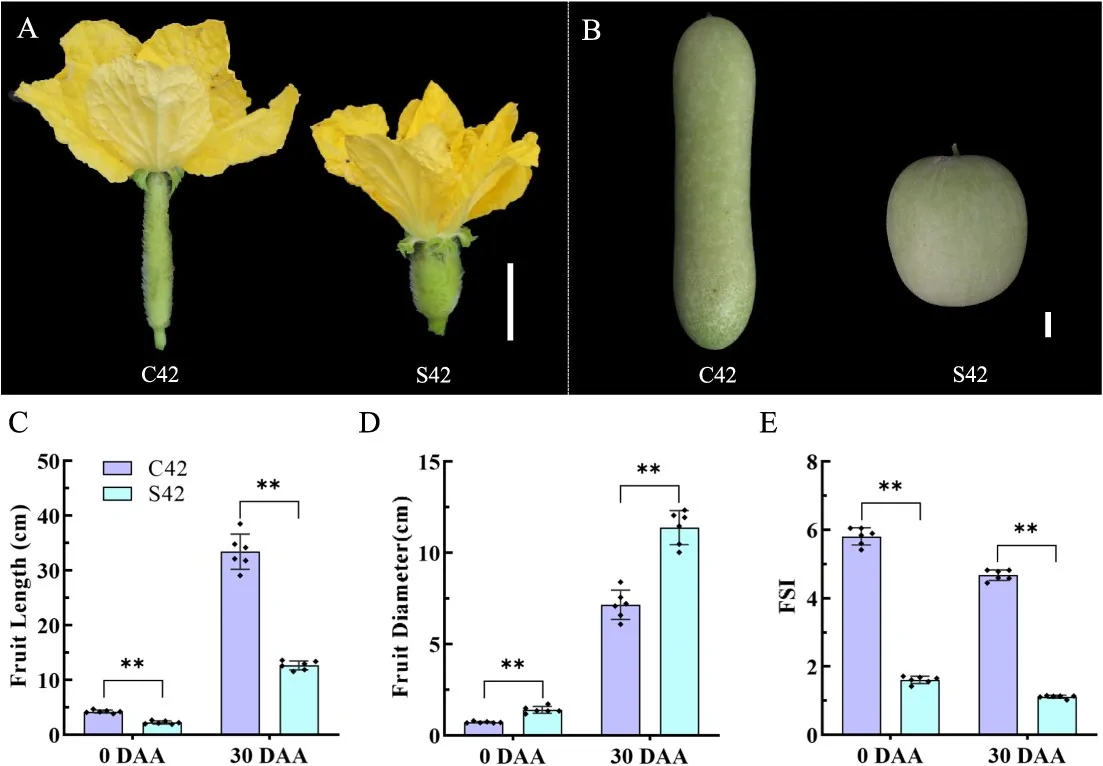
Fruit phenotype divergence between NILs C42 and S42. (A) Representative female flowers of C42 and S42 at 0 DAA. (B) Representative mature fruits of C42 and S42 at 30 DAA. (C–E) Statistical analysis of FL, FD, and FSI for C42 and S42 at 0 and 30 DAA. Data are presented as means ± SE, with individual data points shown as dots. Asterisks indicate significant differences between C42 and S42 (^**^P < 0.01, Student’s *t*-test; *n* = 6). Scale bars in (A) and (B) = 2 cm.

To validate the contribution of natural variation in *BhSUN* to fruit-shape determination, genotyping of the RIL population identified two heterozygous lines, R42 and R87 (Fig. 1D, Table S1). After selfing and genetic fixation, two pairs of near-isogenic lines (NILs), C42/S42 and C87/S87, were developed. In both NIL sets, C42 and C87 produced long cylindrical fruits and carried the *BhSUN^CC–TT^* genotype, whereas S42 and S87 produced spherical fruits and carried the *BhSUN^GG–GG^* genotype (Fig. 2A and B, Fig. S3A and B).

FL, FD, and the FSI were measured at 0 days after anthesis (DAA) and 30 DAA in both NIL pairs (Fig. 2C–E, Fig. S3C–E). Compared with C42, S42 exhibited pronounced longitudinal shortening and radial thickening at both developmental stages, accompanied by a marked reduction in FSI. Specifically, FL decreased by 46.78% and 62.10%, FD increased by 92.42% and 58.97%, and FSI decreased by 72.30% and 76.16% at 0 and 30 DAA, respectively. Comparable phenotypic trends were observed in the C87/S87 NIL pair. These results indicate that natural allelic variation at *BhSUN* is a major determinant of fruit-shape divergence in wax gourd, with pronounced differences already evident at the ovary stage. Notably, we observed similar fruit-shape differences at even earlier stages of ovary development (Fig. S4).

**Figure 3:**
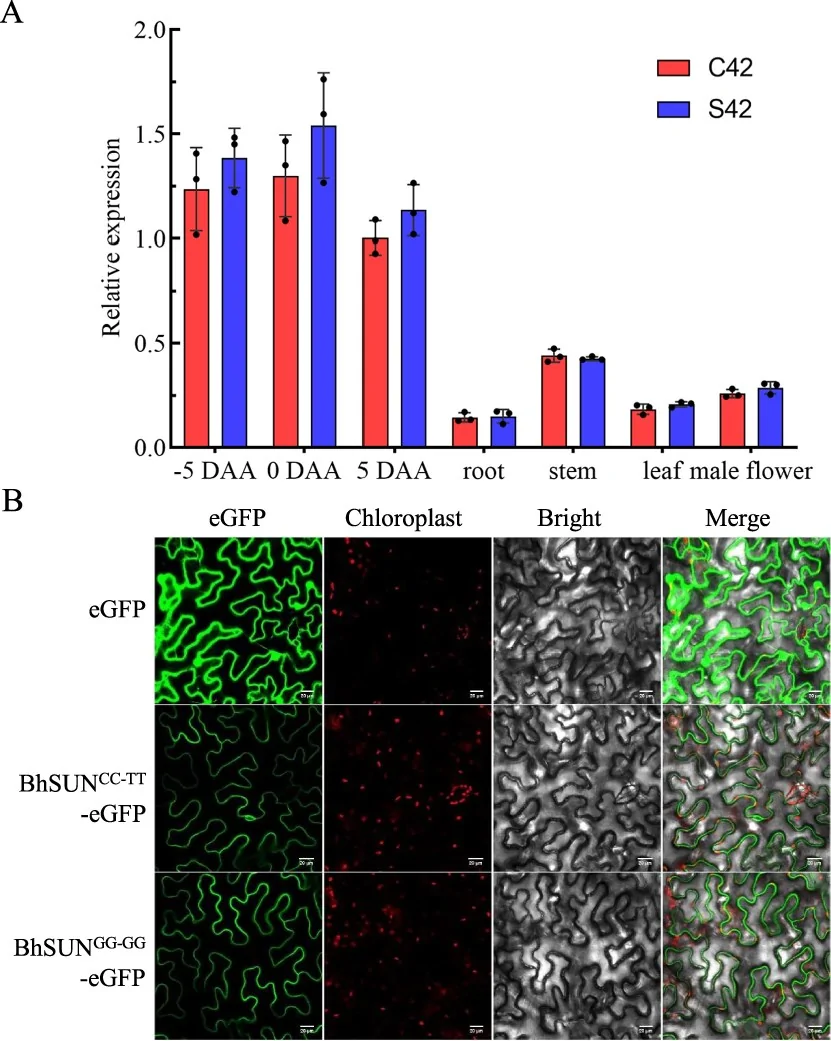
Expression pattern and subcellular localization of *BhSUN*. (A) Relative expression levels of *BhSUN* in C42 and S42. No significant differences were observed between the two genotypes at either time point. Data represent mean ± SD (Student’s *t*-test; *n* = 3). (B) Subcellular localization of eGFP, BhSUN^CC–TT^-eGFP, and BhSUN^GG–GG^-eGFP in leaf epidermal cells. Green fluorescence indicates eGFP signal, red fluorescence indicates chloroplast autofluorescence, and bright-field images show cell structure. The merged images demonstrate that both BhSUN variants are predominantly localized to the plasma membrane. Scale bars = 20 μm.

To investigate the expression pattern of *BhSUN*, we quantified its transcript levels in two inbred lines, C42 and S42, at −5, 0, and 5 DAA. No significant differences in *BhSUN* expression were detected between the two lines at any of the examined developmental stages. Reverse transcription quantitative polymerase chain reaction (RT-qPCR) analysis across various tissues further revealed that *BhSUN* is predominantly expressed in fruit. In addition, transient expression assays showed that the 35S:BhSUN-eGFP fusion protein is mainly localized to the plasma membrane, indicating that the mutation in *BhSUN* does not affect its subcellular localization or overall expression level (Fig. 3A and B).

### Genetic validation of *BhSUN* effects and marker–trait co-segregation analysis

To determine the genetic behavior of *BhSUN*, fruit shape was evaluated in F₂ populations derived from C42 × S42 and C87 × S87 crosses (Table S3). F₁ plants from both crosses produced cylindrical fruits. In the C42 × S42 F₂ population, long cylindrical, cylindrical, and spherical fruits segregated in an approximately 1:2:1 ratio (94:188:102), consistent with the segregation of a single semidominant locus (*χ^2^* = 0.500, *P* = .779). A similar segregation pattern was observed in the C87 × S87 F₂ population (93:201:90; *χ^2^* = 0.891, *P* = .641). These results indicate that wax gourd fruit shape in these populations is primarily controlled by a single semidominant locus.

To further assess marker–trait association, a kompetitive allele-specific PCR marker, SUN-2, was developed based on an A/C polymorphism in the complementary strand of the *BhSUN* locus (corresponding to a T/G polymorphism in the reference strand; Table S4). Genotyping of 768 F₂ individuals from two NIL-derived populations revealed complete co-segregation between SUN-2 genotypes and fruit-shape phenotypes (Fig. S5A, Table S5), further supporting *BhSUN* as the causal determinant underlying fruit-shape variation.

### *BhSUN* deficiency drives the conversion of fruit shape from long cylindrical to spherical

**Figure 4:**
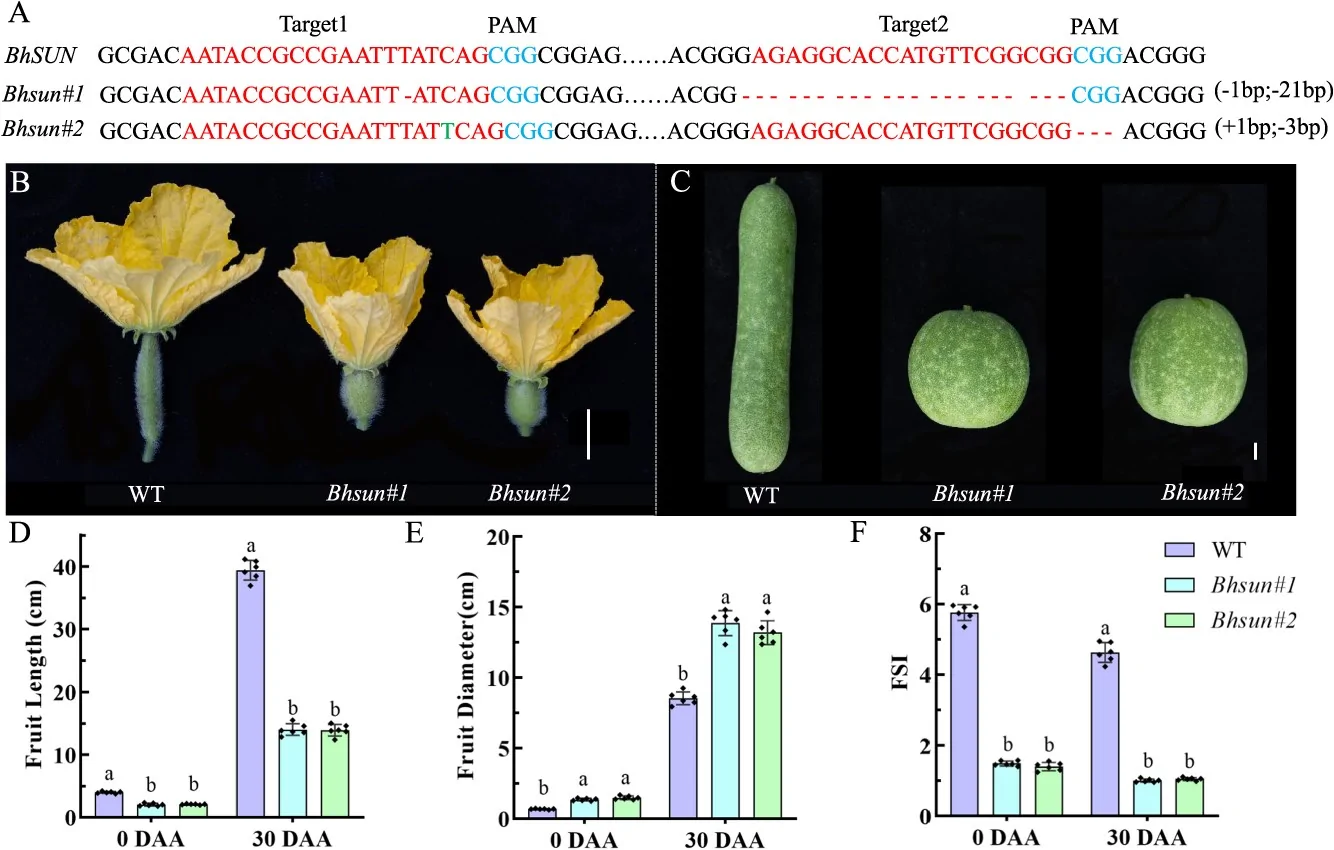
Phenotypic characterization of WT and *Bhsun* mutant plants. (A) Sequence alignment of CRISPR/Cas9 target sites in *BhSUN* showing mutations in *Bhsun* knockout lines. Two sgRNA target sites (Target1 and Target2) and their adjacent protospacer adjacent motif (PAM) sequences are indicated. Insertions, deletions, and PAM sequences are labeled in the sequence alignment. (B) Representative female flowers of WT, *Bhsun#1*, and *Bhsun#2* at 0 DAA. (C) Representative mature fruits of WT, *Bhsun#1*, and *Bhsun#2* at 30 DAA. (D–F) Statistical analysis of FL, FD, and FSI at 0 and 30 DAA. Data are presented as means ± SE, with individual data points shown as dots. Different lowercase letters indicate significant differences (*P* < 0.05, Tukey’s test; *n* = 6). Scale bars in (B) and (C) = 2 cm.

To further validate the function of *BhSUN*, CRISPR/Cas9-mediated genome editing was performed in the long-cylindrical-fruit background TL-8. Two sgRNAs targeting exon 1 were designed (Fig. S5B), and sequencing of T₁ plants identified two independent homozygous null alleles, designated *Bhsun#1* and *Bhsun#2* (Fig. 4A, Fig. S6).

At 30 DAA, FL in *Bhsun#1* and *Bhsun#2* was reduced by 64.48% and 64.72%, respectively, compared with the wild type (WT), whereas FD increased by 62.54% and 54.56%. Accordingly, the FSI declined sharply from 4.62 in WT to 1.01 and 1.05 in the two mutants, resulting in a pronounced spherical fruit phenotype (Fig. 4D–F). These results indicate that loss of *BhSUN* function markedly alters both longitudinal and transverse fruit growth, thereby redirecting fruit development toward a spherical morphology and confirming the essential role of *BhSUN* in regulating fruit shape in wax gourd.

### *BhSUN* regulates cell division pattern and cell expansion

**Figure 5:**
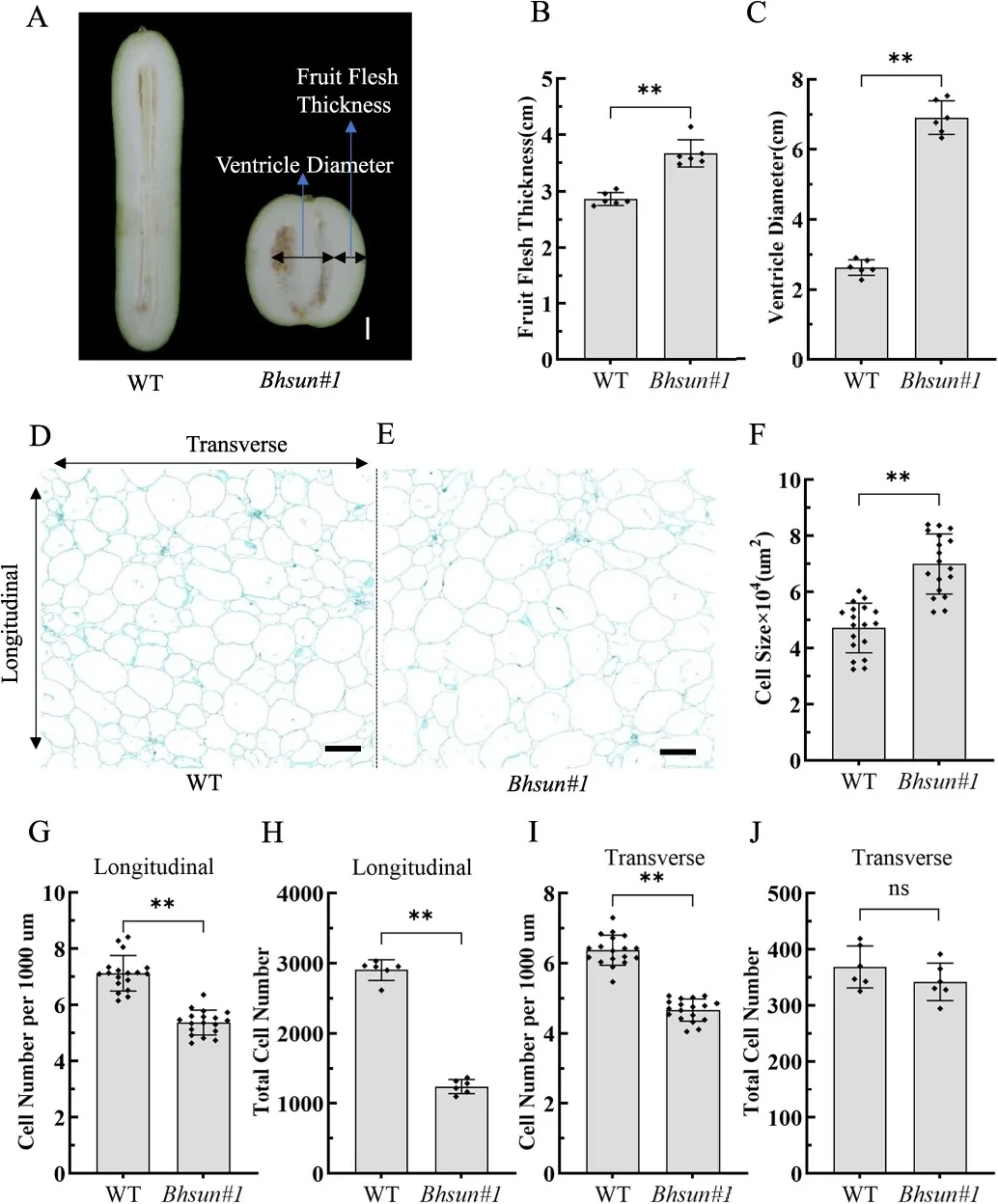
Altered cell expansion and cell division patterns in the mesocarp of *Bhsun#1*. (A) Representative longitudinal sections of mature fruits from WT and *Bhsun#1* at 30 DAA. (B and C) Quantification of fruit flesh thickness and ventricle diameter in WT and *Bhsun#1* fruits. (D and E) Paraffin sections of mesocarp tissue from WT and *Bhsun#1* fruits at 30 DAA. Arrows indicate longitudinal and cross directions. (F) Quantification of mesocarp cell size. (G and H) Number of mesocarp cells per 1000 μm and estimated total cell number along the longitudinal axis. (I and J) Number of mesocarp cells per 1000 μm and estimated total cell number along the cross axis. Data are presented as means ± SE. Asterisks indicate significant differences (^**^P < 0.01, Student’s *t*-test; *n* = 6); ns, not significant. Scale bars: (A) = 2 cm; (D and E) = 200 μm.

To investigate the histological basis of the spherical fruit phenotype caused by *BhSUN* disruption, internal fruit structures were compared between WT and mutant plants. In *Bhsun#1*, mesocarp thickness and locule diameter increased to 1.28-fold and 2.63-fold of WT levels, respectively, indicating that mesocarp thickening and locule enlargement jointly contribute to enhanced transverse fruit growth (Fig. 5A–C). To further elucidate the cellular basis underlying changes in the FSI, mesocarp cell organization was quantified along both the longitudinal and transverse axes. In fruits at 30 DAA, the number of mesocarp cells per unit length was significantly lower in *Bhsun#1* than in WT along the longitudinal axis (WT: 7.12 ± 0.61; *Bhsun#1*: 5.37 ± 0.42) as well as the transverse axis (WT: 6.37 ± 0.41; *Bhsun#1*: 4.66 ± 0.30) (Fig. 5D and E).

From a morphological approximation, long cylindrical wax gourd fruits can be regarded as cylindrical, with mesocarp length corresponding closely to longitudinal FL, whereas spherical fruits can be approximated as spheres, with mesocarp length roughly equivalent to half of the fruit circumference. Based on this framework, the estimated total number of longitudinal mesocarp cells was markedly lower in *Bhsun#1* than in WT (1242.48 ± 91.95 vs. 2903.34 ± 134.00), indicating strong suppression of longitudinal cell proliferation. In contrast, the estimated total number of transverse cells did not differ significantly between *Bhsun#1* and WT (341.88 ± 30.56 vs. 368.56 ± 34.32) (Fig. 5G–J), suggesting that transverse cell proliferation is largely maintained. In addition, mesocarp cell area was significantly increased in *Bhsun#1* relative to WT (Fig. 5F), indicating enhanced cell expansion in the mutant. Collectively, these results suggest that *BhSUN* modulates wax gourd fruit morphology through coordinated regulation of cell division pattern and the extent of cell expansion.

### *BhSUN* directly interacts with Ca^2+^-sensor proteins and the microtubule-associated protein

To delineate the regulatory network downstream of *BhSUN*, transcriptome profiling was conducted on fruits sampled at 0 DAA from C42 vs. S42 and WT vs. *Bhsun#1*. Differential expression analysis identified 411 differentially expressed genes (DEGs) in C42 vs. S42 and 1874 DEGs in WT vs. *Bhsun#1*. Intersection of the two DEG datasets yielded 224 shared genes, which likely constitute a core *BhSUN*-responsive transcriptional module (Table S6).

**Figure 6:**
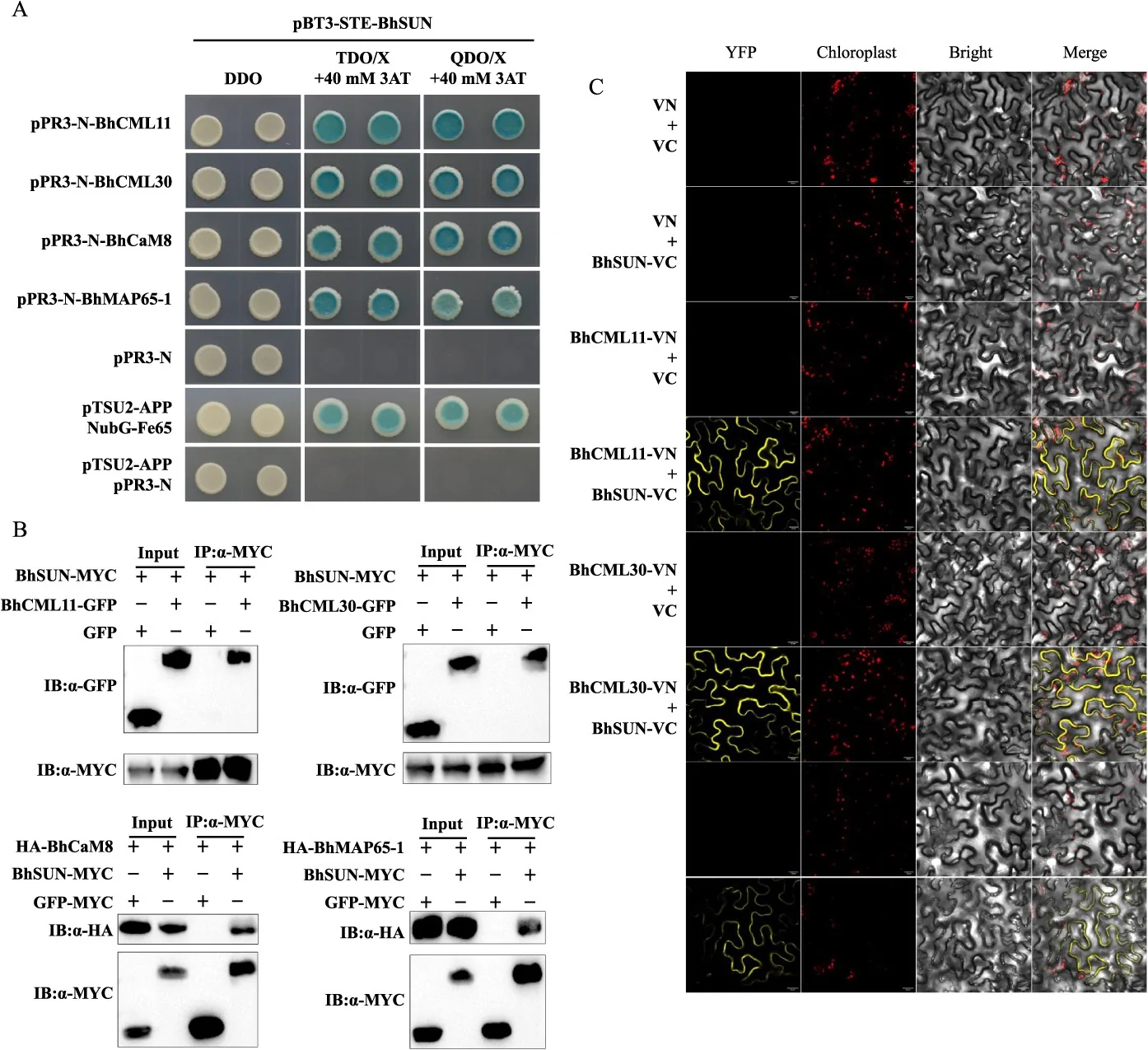
BhSUN interacts with CaM/CML and MAP65 proteins *in vitro* and *in planta*. (A) Y2H assays showing the interactions between BhSUN and BhCML11, BhCML30, BhCaM8, and BhMAP65-1. The pBT3-STE-BhSUN construct was co-transformed with the corresponding pPR3-N vectors into yeast cells. Transformants were grown on DDO (SD/−Leu/−Trp), TDO/X (SD/−Leu/−Trp/−His supplemented with 40 mM 3-AT and X-α-gal), and QDO/X (SD/−Leu/−Trp/−His/−Ade supplemented with 40 mM 3-AT and X-α-gal) media. Colony growth and reporter-gene activation indicate positive protein–protein interactions. pTSU2-APP/NubG-Fe65 was used as a positive control, and empty vector combinations served as negative controls. (B) Co-IP assays confirming the *in vivo* interactions between BhSUN and BhCML11, BhCML30, BhCaM8, and BhMAP65-1. Tagged proteins were transiently co-expressed in *N. benthamiana* leaves. Total protein extracts (Input) were immunoprecipitated with an anti-MYC antibody (IP: α-MYC), followed by IB using anti-GFP, anti-HA, or anti-MYC antibodies as indicated. (C) BiFC assays showing the interactions of BhSUN with BhCML11, BhCML30, BhCaM8, and BhMAP65-1 in *N. benthamiana* epidermal cells. BhSUN was fused to VC, while BhCML11, BhCML30, BhCaM8, or BhMAP65-1 was fused to VN. Reconstituted fluorescence signals indicate protein–protein interactions. Bright-field images and merged images are shown on the right. VN + VC and single-vector combinations were used as negative controls. Scale bars = 20 μm.

Given that IQD proteins are known to bind CaM complexes *via* conserved motifs, three CaM/CML family genes were identified among the 224 shared DEGs (Fig. S7): *Bch01G006510* (*BhCML11*), *Bch04G003080* (*BhCML30*), and *Bch12G015250* (*BhCaM8*). Yeast two-hybrid (Y2H) assays revealed that BhSUN physically interacts with the three CaM/CML proteins (Fig. 6A). These interactions were further confirmed by co-immunoprecipitation (Co-IP) in *Nicotiana benthamiana*, demonstrating association in a plant cellular environment (Fig. 6B). Bimolecular fluorescence complementation (BiFC) assays showed that the BhSUN–CaM/CML complexes specifically reconstitute fluorescence at the plasma membrane, supporting their colocalization *in vivo* (Fig. 6C). Together, these results provide strong evidence for the formation of BhSUN–CaM/CML complexes at the cell membrane, suggesting a potential role in calcium-mediated signaling during fruit-shape regulation.

MTs play a central role in cell plate orientation and organ morphogenesis. In tomato, *SlSUN18* has been shown to interact with the MT-associated protein SlMAP65 to modulate cell proliferation patterns and fruit shape. In wax gourd, expression profiling of MAP65 family members revealed that *Bch11G005560* (*BhMAP65-1*) was highly expressed in the ovary at 0 DAA (Fig. S7). Protein–protein interaction assays, including Y2H, Co-IP, and BiFC, confirmed a direct interaction between BhSUN and BhMAP65-1 (Fig. 6A–C). Specifically, membrane-based Y2H assays indicated that BhSUN and BhMAP65-1 interact at the plasma membrane in yeast; this was corroborated by Co-IP in *N. benthamiana*, which demonstrated their physical association in planta. Consistently, BiFC analysis also revealed reconstituted fluorescence signals at the plasma membrane, confirming that the BhSUN–BhMAP65-1 complex forms *in vivo* at the cell periphery. Collectively, these results suggest that BhSUN may integrate Ca^2+^ signaling with MT dynamics through coordinated interactions with CaM/CML and MAP65 proteins, thereby contributing to fruit growth and morphogenesis.

### *BhSUN*-associated fruit-shape divergence is accompanied by phytohormone metabolic reprogramming

**Figure 7:**
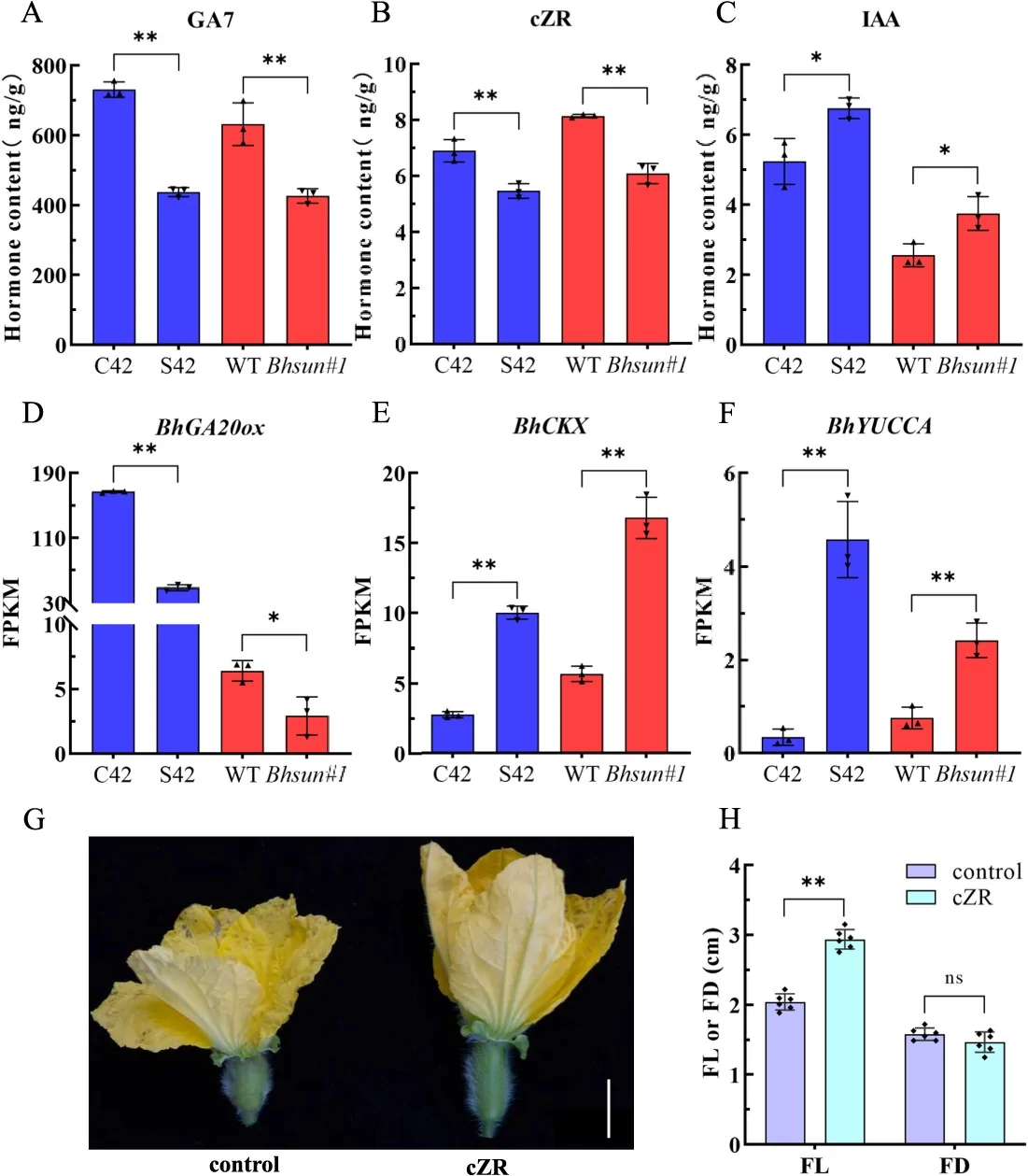
Association between phytohormone dynamics and fruit-shape variation in wax gourd. (A–C) Endogenous levels of GA7, cZR, and IAA in fruits of C42, S42, WT, and *Bhsun#1* at 0 DAA (*n* = 3). (D–F) Expression levels (FPKM) of hormone metabolism-related genes *BhGA20ox*, *BhCKX*, and *BhYUCCA* in C42, S42, WT, and *Bhsun#1* (*n* = 3). (G) Representative fruit phenotypes at 0 DAA under control conditions and after exogenous cZR treatment. (H) Effects of cZR treatment on FL and FD at 0 DAA (*n* = 3). Data are presented as means ± SE. Asterisks indicate significant differences (^*^P < 0.05; ^**^P < 0.01, Student’s *t*-test); ns, not significant. Scale bar in (G) = 2 cm.

Phytohormones play central roles in coordinating cell division and cell expansion during fruit development. To assess the hormonal contributions to *BhSUN*-mediated fruit-shape divergence, targeted phytohormone metabolomic profiling using liquid chromatography–mass spectrometry (LC–MS) was performed on fruits sampled at 0 DAA from C42 vs. S42 and WT vs. *Bhsun#1*. In total, 22 hormone-related metabolites were detected, encompassing the abscisic acid, auxin, gibberellin, and cytokinin pathways (Table S7). Comparative analysis identified nine and eight differentially accumulated metabolites in C42 vs. S42 and WT vs. *Bhsun#1*, respectively. Among these, three metabolites—gibberellin A7 (GA7), *cis*-zeatin riboside (cZR), and indole-3-acetic acid (IAA)—exhibited consistent changes in both comparisons (Fig. 7A–C), implicating them as key hormonal components associated with fruit-shape divergence.

Integration of metabolomic and transcriptomic datasets further revealed differential expression of hormone-related genes among the shared *BhSUN*-responsive DEGs. *Bch04G001820* (*BhCKX*), encoding a cytokinin oxidase/dehydrogenase, and *Bch05G001090* (*BhYUCCA*), encoding an indole-3-pyruvate monooxygenase involved in auxin biosynthesis, were upregulated in spherical fruits. In contrast, *Bch09G009730* (*BhGA20ox*), encoding gibberellin 20-oxidase, showed higher expression in long cylindrical fruits (Fig. 7D–F). These results indicate that coordinated reprogramming of cytokinin, auxin, and gibberellin metabolism is associated with wax gourd fruit-shape determination.

To further evaluate the functional relevance of cZR, exogenous cZR was applied to *Bhsun#1* plants. Treated fruits exhibited significantly increased FL compared with control plants (Fig. 7G and H), supporting a role for cZR in promoting longitudinal growth and its involvement in *BhSUN*-mediated fruit-shape regulation.

## Discussion

*SUN* genes, which encode IQD proteins, play a conserved role in shaping organ morphology across plant species. In cucumber, nonsynonymous mutations in *CsSUN* result in reduced FL and increased FD, with amino acid substitutions within the IQ domain proposed to underlie functional alteration. Consistently, knockout of *CsSUN* produces phenotypes indistinguishable from natural mutant alleles, indicating that these mutations directly impair gene function [11]. By contrast, fruit-shape variation associated with *ClSUN* in watermelon is largely attributed to altered gene expression caused by structural variation, while in melon and tomato, *SUN* gene dosage and expression levels strongly influence fruit elongation [21, 22]. In the present study, we identified a naturally occurring nonsynonymous mutation within the conserved IQ domain of *BhSUN* (Fig. 1, Fig. S6), and CRISPR/Cas9-mediated knockout further demonstrated that loss of *BhSUN* function results in a dramatic transition from long cylindrical to spherical fruits (Fig. 4). Together, these findings indicate that both natural alleles and targeted mutations lead to functional loss of *BhSUN* activity, and further suggest that variation within the conserved IQ domain represents a key molecular mechanism underlying SUN protein functional divergence. More broadly, these results highlight *BhSUN* as a critical functional node linking conserved IQD protein structure to fruit-shape diversification in cucurbit crops.

Final fruit morphology is determined by the rate, duration, and orientation of cell division, together with anisotropic cell expansion. Previous studies have shown that SUN family genes regulate fruit shape through distinct cellular mechanisms across species [23, 24]. For example, *SlSUN* primarily modulates cell division patterns in tomato, whereas *CsSUN* in cucumber influences both cell division and cell expansion, and *CaSUN29* in pepper predominantly affects cell expansion [11, 12, 25, 26]. In agreement with these findings, our histological analyses demonstrate that *BhSUN* regulates both the orientation of cell division and the extent of cell expansion (Fig. 5). Specifically, loss of *BhSUN* function results in reduced longitudinal cell proliferation and enhanced radial cell expansion, thereby altering the longitudinal-to-transverse growth ratio and ultimately leading to spherical fruit morphology. These results indicate that *BhSUN* promotes anisotropic growth along the longitudinal axis and suggest that SUN-mediated regulation of organ shape is conserved yet exhibits species-specific diversification in its cellular implementation.

Ca^2+^ signaling plays a central role in plant morphogenesis, and IQD proteins are widely recognized as key decoders of Ca^2+^-CaM signaling [27, 28]. Previous studies have shown that IQD proteins interact with CaM and MT-associated proteins to regulate MT organization and cell elongation [17, 29–31]. Consistent with this framework, we demonstrate that BhSUN interacts with multiple CaM/CML proteins as well as the MT-associated protein BhMAP65-1 (Fig. 6). These interactions suggest that BhSUN may form a protein complex at the plasma membrane that integrates Ca^2+^ signaling with MT dynamics. Given the essential role of MTs in determining the orientation of cell division and expansion, disruption of this regulatory module provides a mechanistic explanation for the altered growth patterns observed in Bhsun mutants. Collectively, these findings indicate that BhSUN functions as a central molecular hub that links Ca^2+^ signal decoding with MT organization to coordinate cell division patterns and cell expansion during fruit development.

Phytohormones exert hierarchical control over cell proliferation and cell expansion during fruit development [32]. For example, auxin promotes cell expansion, whereas cytokinin regulates cell division, and gibberellins contribute to both processes [33–37]. In this study, we observed coordinated changes in auxin, cytokinin, and gibberellin pathways associated with *BhSUN*-mediated fruit-shape divergence (Fig. 7). Spherical fruits exhibited elevated IAA levels and increased cell size, suggesting that altered auxin accumulation contributes to enhanced radial expansion. In addition, *BhCKX* expression was significantly upregulated in spherical fruits, accompanied by reduced cytokinin levels, while exogenous application of cZR promoted fruit elongation (Fig. 7), supporting a role for cytokinin in longitudinal growth. Together, these results suggest that *BhSUN* may regulate fruit shape by modulating hormone homeostasis, thereby influencing the balance between cell division and cell expansion. However, whether *BhSUN* directly regulates hormone biosynthesis and signaling genes or indirectly affects hormone distribution through MT dynamics remains to be determined.

**Figure 8:**
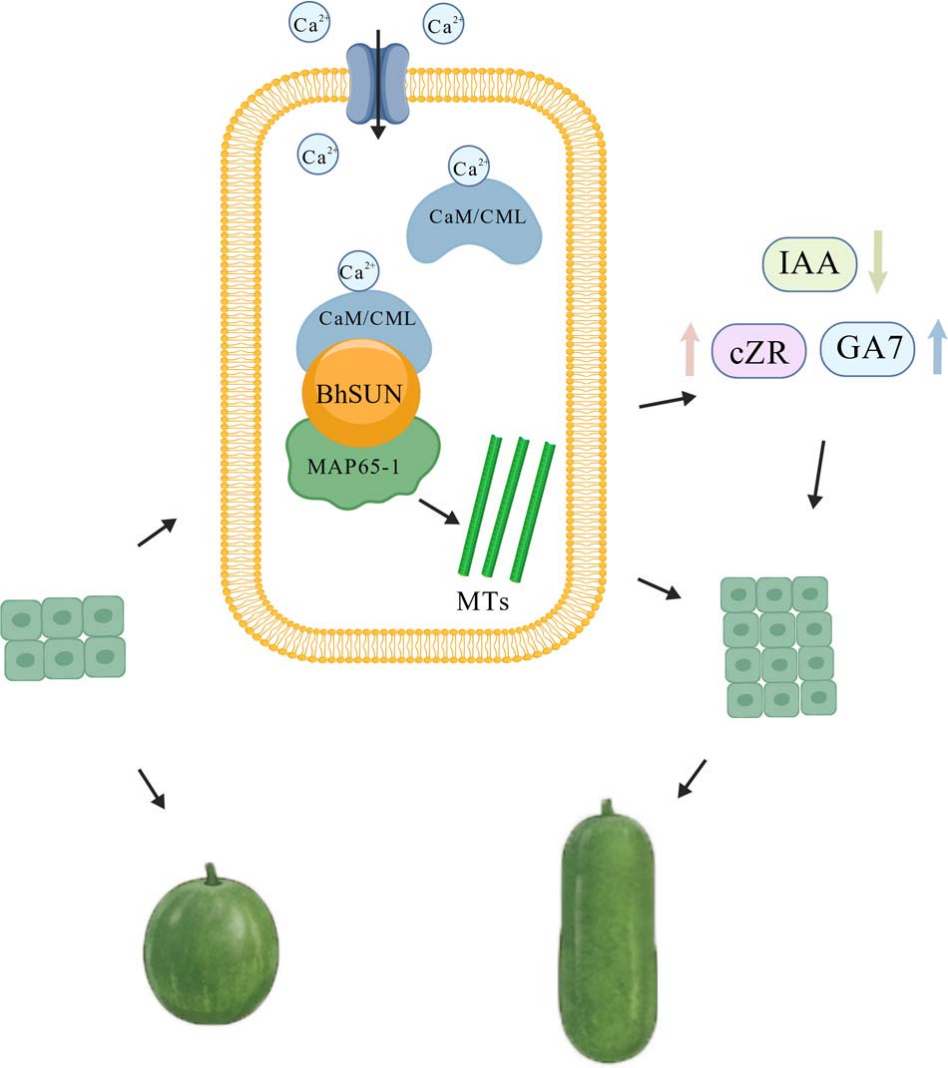
Proposedllllllllllmodel for BhSUN-mediated regulation of fruit shape in wax gourd. External or intracellular signals induce Ca^2+^ influx, which is sensed by CaM/CML. These proteins interact with BhSUN to form a signaling complex. BhSUN then associates with the MT-associated protein BhMAP65-1, regulating the organization and dynamic stability of MTs, which in turn affects the orientation of cell division planes and the pattern of cell expansion. Meanwhile, reprogramming of the hormone homeostasis of IAA, cZR, and GA7 coordinates the intensity of cell proliferation and expansion. The combined effects of these regulatory pathways ultimately determine cell arrangement and tissue morphology, leading to changes in fruit shape (such as the transition from spherical to elongated forms).

Taken together, our findings support a model in which *BhSUN* coordinates calcium signaling, MT dynamics, and phytohormone homeostasis to regulate directional fruit growth. In this framework, *BhSUN*-mediated integration of Ca^2+^ signaling and MT organization governs the orientation of cell division and anisotropic cell expansion, while concomitant modulation of hormone pathways further fine-tunes growth patterns during early fruit development. The convergence of these regulatory processes ultimately determines the balance between longitudinal and radial growth, leading to distinct fruit shapes (Fig. 8).

Despite these advances, several important questions remain unresolved. The structural basis by which nonsynonymous mutations disrupt *BhSUN* function, as well as the causal relationship between *BhSUN* activity and hormone regulation, require further investigation. Given that BhSUN interacts with CaM/CML proteins and BhMAP65-1, it is possible that amino acid substitutions impair the formation or stability of these protein complexes. In addition, whether *BhSUN* directly regulates hormone metabolism or indirectly influences hormone distribution through MT organization remains unclear. Future studies integrating structural biology, genetic interaction analysis, and high-resolution cellular imaging will be essential to fully elucidate the mechanistic framework underlying *BhSUN*-mediated fruit morphogenesis.

## Materials and methods

### Plant materials

A total of 105 RILs developed for QTL analysis were grown at the experimental field of Guangxi University during autumn 2022 and spring 2023. For each RIL, three representative plants were selected for phenotypic evaluation. FL and FD were measured at the commercial maturity stage, and the FSI was calculated as the ratio of FL to FD. Two pairs of NILs, C42/S42 and C87/S87, were developed from heterozygous RILs. F₁ plants were generated by crossing each NIL pair and subsequently self-pollinated to produce F₂ populations. Fruit shape-related traits were recorded for a total of 768 F₂ individuals derived from the two crosses.

### QTL mapping

Whole-genome resequencing was performed for all 105 RILs, and high-quality clean reads were aligned to the wax gourd reference genome. A high-density genetic linkage map previously constructed by was used for QTL analysis [38, 39]. Composite interval mapping was conducted using QTL Cartographer to identify loci associated with FSI. The significance threshold for QTL detection was determined by 1000 permutation tests at a confidence level of *P* = 0.05.

### Expression analysis

Samples were collected from C42 and S42 at each stage, with three biological replicates per germplasm, and were immediately frozen in liquid nitrogen for subsequent analysis. Total RNA was extracted using a Promega RNA extraction kit and evaluated for concentration, purity, and integrity. cDNA was synthesized using Takara RT Master Mix. Gene-specific primers were designed with NCBI Primer-BLAST. RT-qPCR was performed on a QuantStudio 6 Flex system using Takara SYBR Green reagents, with Actin (*Bch10G018990*) as the internal reference. Relative expression levels were calculated using the 2^−ΔΔCT^ method.

### Subcellular localization analysis

The coding sequence (CDS) of *BhSUN* without the stop codon was amplified from C42 and cloned into the pBI121-eGFP vector to generate the 35S:BhSUN-eGFP fusion construct. The 35S:eGFP empty vector was used as a control. Recombinant plasmids were introduced into *Agrobacterium tumefaciens* strain GV3101 and transiently expressed in the abaxial epidermal cells of *N. benthamiana* leaves by *Agrobacterium*-mediated infiltration. After incubation for 2 days under standard growth conditions, fluorescence signals were observed using a confocal laser scanning microscope. Images were processed using ImageJ software.

### CRISPR/Cas9-mediated gene editing in wax gourd

Targeted mutagenesis of *BhSUN* was performed in the long-cylindrical-fruit wax gourd line TL-8 using the CRISPR/Cas9 system. Two sgRNAs were designed using CRISPR-P to target the first exon of BhSUN, and were assembled into the gene-editing vector. Wax gourd transformation was carried out using a protocol adapted from established cucumber transformation methods [40]. Briefly, the CRISPR/Cas9 construct was introduced into *A. tumefaciens* strain EHA105 and verified by PCR. Cotyledons excised from 2-day-old germinated seedlings were used as explants (approximately 150 explants per batch) and infected with *Agrobacterium* suspension for 30 min at room temperature, with the total handling time not exceeding 2 h. Explants were cocultivated in the dark at 23°C for 3 days, transferred to resting medium (25°C, 7 days under light), and then moved to selection medium containing spectinomycin for approximately 6 weeks to induce resistant shoots. Subsequently, the regenerated shoots were transferred to elongation medium and cultured for 3–6 weeks until rooting. Putative transformants were initially identified based on green fluorescent protein (GFP) fluorescence and further confirmed by sequencing to obtain T₀ plants. The T₀ plants were then self-pollinated to generate the T₁ generation, from which homozygous mutants were selected by sequencing for subsequent phenotypic analysis.

### Histological analysis

Fruits were sampled along the longitudinal axis and immediately fixed in formalin–acetic acid–alcohol (FAA) solution. After fixation, samples were dehydrated through a graded ethanol series, cleared with xylene, and embedded in paraffin. Longitudinal sections (10 μm) were prepared using a rotary microtome and observed under a light microscope. Cell area and cell number were quantified using ImageJ software.

### RNA sequencing analysis

Fruits were harvested at 0 DAA for transcriptome profiling, with three biological replicates per sample. RNA library preparation and sequencing were performed using the DNBSEQ-T7 platform. After quality filtering, clean reads were aligned to the wax gourd reference genome using HISAT2 [41]. DEGs were identified using DESeq2, with thresholds of | log₂ (fold change) | > 1 and adjusted *P*-value <0.05 [42].

### Yeast two-hybrid assay

The full-length CDS of *BhSUN* was cloned into the bait vector pBT3-STE, whereas the full-length CDSs of *BhCML11*, *BhCML30*, *BhCaM8*, and *BhMAP65-1* were cloned into the prey vector pPR3-N. The pTSU2-APP/pNubG-Fe65 pair was used as a positive control, and the empty pPR3-N vector served as a negative control. Bait and prey constructs were co-transformed into the yeast strain NMY51. Transformants were sequentially plated on DDO, TDO/X supplemented with 40 mM 3-amino-1,2,4-triazole (3-AT), and QDO/X supplemented with 40 mM 3-AT, followed by incubation at 30°C for 3–5 days to assess protein–protein interactions.

### Bimolecular fluorescence complementation assay

The full-length CDS of *BhSUN* was cloned into the pGreenII-62-SK-VC vector, while the full-length CDSs of *BhCML11*, *BhCML30*, *BhCaM8*, and *BhMAP65-1* were individually inserted into the pGreenII-62-SK-VN vector. The resulting recombinant plasmids were introduced into *A. tumefaciens* strain GV3101-P19. Co-infiltration assays were carried out by syringe-injecting mixtures of agrobacterial cultures [containing paired C-terminal fragment of Venus (VC) and N-terminal fragment of Venus (VN) constructs] into the abaxial side of *N. benthamiana* leaves. Following infiltration, plants were incubated under low-light conditions for 2 days. Leaf sections from the infiltrated areas were then excised, mounted on microscope slides, and examined under a confocal laser scanning microscope to detect fluorescence complementation signals indicative of protein–protein interactions.

### Co-immunoprecipitation assay

The full-length CDS of *BhSUN* was cloned into the pBWA(V)H2STMVΩ vector to generate a BhSUN-MYC fusion construct. Constructs expressing BhCML11-GFP, BhCML30-GFP, HA-BhCaM8, and HA-BhMAP65-1 were generated accordingly. The BhSUN-MYC construct and candidate interactor constructs were co-introduced into *A. tumefaciens* strain GV3101 and transiently co-expressed in *N. benthamiana* leaves by *Agrobacterium*-mediated infiltration. After 2 days of growth under greenhouse conditions, infiltrated leaves were harvested, immediately frozen in liquid nitrogen, and ground to a fine powder. Total proteins were extracted using lysis buffer supplemented with protease inhibitors. Input samples were denatured with 4× SDS loading buffer, whereas immunoprecipitation samples were incubated with antibody-conjugated magnetic beads at 4°C for 16 h. After washing, bound proteins were eluted with 4× SDS loading buffer, separated by sodium dodecyl sulfate–polyacrylamide gel electrophoresis (SDS-PAGE), transferred to membranes, and detected by immunoblotting (IB) using anti-GFP, anti-HA, and anti-MYC antibodies.

### Targeted phytohormone metabolomic analysis

A total of 41 plant hormone standards were dissolved in methanol and serially diluted to generate mixed working solutions. Fresh fruit samples (100 mg) were extracted with 80% methanol containing internal standards, homogenized for 3 min at −20°C, and ultrasonicated for 60 min at 5°C. Supernatants were collected following extraction according to the EN15662 method and subjected to LC–MS/MS analysis [43].

Chromatographic separation was performed using an ExionLC AD system coupled with a QTRAP 6500+ mass spectrometer on a Waters BEH C18 column, with gradient elution over a total runtime of 12 min. Data were acquired in both positive and negative electrospray ionization modes. Quality control samples were injected at regular intervals to monitor instrument stability. Raw data were processed using Sciex OS software, and quantified metabolite data were uploaded to the Majorbio Cloud platform for orthogonal partial least squares discriminant analysis using R packages to identify differentially accumulated metabolites.

### Exogenous *cis*-zeatin riboside treatment

Exogenous cZR treatment was applied to *Bhsun#1* plants. The cZR concentration was determined based on preliminary experiments, and foliar spraying was initiated at the 10-leaf stage using distilled water (control) or 50 mg l^−1^ cZR. Each treatment included three biological replicates, with 10 plants per replicate. The shoot apex and the three youngest fully expanded leaves immediately below the apex were sprayed until uniformly moistened. A second application was performed 7 days after the first treatment using the same procedure.

### Statistical analyses

Statistical analyses were performed using IBM SPSS Statistics 24. Differences between experimental groups were assessed using one-way analysis of variance and independent-samples *t*-tests, as appropriate.

## Supplementary Material

Web_Material_uhag190

## Data Availability

The raw sequencing data have been deposited in the China National Center for Bioinformation National Genomics Data Center (https://ngdc.cncb.ac.cn/) under accession number CRA036008.
