# Supplementary material for: BhSUN coordinates calcium signaling, microtubule organization, and hormone metabolism to drive directional fruit growth in wax gourd
Source: Hortic Res. 2026 May 12;13(10):uhag190. doi: 10.1093/hr/uhag190 (PMC13616496; doi:10.1093/hr/uhag190)
Supplement: Supplementary file 1 [file web_material_uhag190.zip › Supplementary Figures.docx]

**Figure S1.** Phylogenetic analysis of BhSUN and SUN proteins from tomato. A phylogenetic tree was constructed based on the amino acid sequences of BhSUN from wax gourd and 33 SUN proteins from tomato. BhSUN clusters closely with SlSUN18 (highlighted by a red box), indicating a close evolutionary relationship between the two proteins. Numbers at the nodes represent bootstrap support values (%).

**Figure S2.** Multiple sequence alignment of BhSUN and its homologous proteins. Amino acid sequences of BhSUN homologs from different plant species were aligned. Red arrows indicate the positions of amino acid substitutions identified in BhSUN.

**Figure S3.** Fruit phenotype divergence between near-isogenic lines C87 and S87. (A) Representative female flowers of C87 and S87 at 0 days after anthesis (DAA). (B) Representative mature fruits of C87 and S87 at 30 DAA. (C) Statistical analysis of FL in C87 (4.25±0.44; 26.51±1.50) and S87 (2.06±0.27; 12.25±0.85) at 0 DAA and 30 DAA. (D) Statistical analysis of FD in C87 (0.72±0.04; 6.26±0.26) and S87 (1.35±0.16; 11.26±0.81) at 0 DAA and 30 DAA. (E) Statistical analysis of FSI in C87 (5.87±0.37; 4.23±0.13) and S87 (1.53±0.11; 1.08±0.04) at 0 DAA and 30 DAA. Data are presented as means ± SE, with individual data points shown as dots. Asterisks indicate significant differences between C87 and S87 (***P* < 0.01, Tukey’s test; n=6). Scale bars in (A) and (B) = 2 cm.

**Figure S4.** Phenotypic differences in ovary fruit between isogenic lines C42 and S42. Scale bars = 2 cm.

**Figure S5.** (A) SNP genotyping based on the complementary-strand polymorphism (A/C) of *BhSUN* in F₂ populations derived from the crosses C42 × S42 (A) and C87 × S87. Individuals homozygous for allele A/A, homozygous for allele C/C, and heterozygous A/C are indicated by red, blue, and green dots, respectively, while undetermined genotypes are marked with crosses.Gene sequence of *BhSUN*. (B) The *BhSUN* gene sequence is shown, where red represents exons, black represents introns, and the sgRNA target sites are highlighted with a yellow background.

**Figure S6.** (A) Comparison chromatograms of *BhSUN* gene sequences between WT and *BhSUN* mutants. (B) Comparison of BhSUN protein sequences among C42, S42, WT, and Bhsun mutants. The BhSUN protein sequences of C42/C87, S42/S87, and WT are intact, whereas Bhsun#1 and Bhsun#2 exhibit different degrees of truncation or sequence alterations. Red arrows indicate the two predicted IQ motif domains in the BhSUN protein. Red triangles denote the two mutation sites in S42/S87, one of which is located within an IQ motif domain.

**Figure S7.** Identification and expression analysis of CaM/CML and MAP65 family genes associated with BhSUN. (A) Expression levels (FPKM values) of *BhCML11*, *BhCML30*, and *BhCaM8* in C42, S42, WT, and Bhsun#1 at 0 DAA. (B) Heatmap showing the expression profiles (FPKM values) of MAP65 family genes in C42, S42, WT, and Bhsun#1. Among the MAP65 family members, *Bch11G005560* (*BhMAP65-1*) exhibits relatively high expression at 0 DAA.
